# Knowledge, Attitudes, and Practices of Family Medicine Residents Regarding Insulin Overbasalization at the Family Medicine Academy of King Saud Medical City

**DOI:** 10.7759/cureus.89338

**Published:** 2025-08-04

**Authors:** Moayyad Almubaraki, Abdulrahman M Elnasieh, Abdulmohsen Alangari

**Affiliations:** 1 Department of Family Medicine, King Saud Medical City, Riyadh, SAU

**Keywords:** family medicine, insulin therapy, management, overbasalization, type 2 diabetes mellitus

## Abstract

Background: Insulin overbasalization, which refers to the excessive use of basal insulin despite achieving target fasting glucose levels, poses significant clinical risks in managing type 2 diabetes mellitus. This practice can lead to adverse outcomes such as increased hypoglycemia, weight gain, and complications in glycemic control, which ultimately affect the patient's quality of life. Studies indicate that approximately 25-30% of patients on insulin therapy may experience overbasalization, highlighting the urgency of addressing this issue. Given their pivotal role in diabetes management, it is essential to assess family medicine residents (FMRs)' knowledge, attitudes, and practices (KAP) regarding insulin titration to ensure optimal patient outcomes.

Objective: This study aims to explore FMRs' KAP to identify gaps and promote evidence-based, patient-centered diabetes care strategies.

Materials and methods: A cross-sectional study using stratified random sampling was conducted among FMRs at King Saud Medical City from June 2024 to April 2025. Data were collected via a validated self-administered questionnaire, which underwent content validation by a panel of experts in family medicine and diabetes management. The questionnaire was then pilot-tested for reliability and clarity before the main study. Data were analyzed using SPSS Statistics version 29.0 (IBM Corp. Released 2022. IBM SPSS Statistics for Windows, Version 29.0. Armonk, NY: IBM Corp.).

Results: A total of 101 FMRs were assessed. Most were female (n=55, 54.5%) and in their first (n=43, 42.6%) and second (n=37, 36.6%) residency years. The majority had no additional qualifications (n=59, 58.4%). More than half (n=55, 54.5%) had never heard of insulin overbasalization, and only 18 (17.8%) were familiar with the concept. Hypoglycemia (n=78, 77.2%) and weight gain (n=50, 49.5%) were the most commonly recognized consequences. Good knowledge was seen in 53 (52.5%) participants. Only 9 (8.9%) were fully confident in managing overbasalization, and 79 (78.2%) had never educated patients about it. Attitude/practice scores were significantly associated with residency level (p = 0.023), but knowledge scores were not (p = 0.137). Logistic regression confirmed residency year as a significant predictor of good practice (OR = 1.74, p = 0.043). A positive correlation between knowledge and practice was also observed (r = 0.339, p = 0.001).

Conclusions: This study revealed limited awareness and confidence among FMRs regarding insulin overbasalization. While knowledge alone was not significantly linked to residency level, more training years predicted better practice. A significant correlation between knowledge and practice highlights the need for early, targeted education to enhance insulin management skills in primary care.

## Introduction

The treatment of type 2 diabetes mellitus (T2DM) is a complex and nuanced endeavor, requiring a tailored approach to each patient's needs. Among the therapeutic options available, insulin therapy stands as a cornerstone, particularly when oral antidiabetic medications fail to maintain optimal glycemic control. Basal insulin is often the preferred initial formulation [1]. However, its titration must be managed meticulously to avoid complications such as hypoglycemia or insulin overbasalization, the latter referring to the excessive reliance on basal insulin to control hyperglycemia without appropriate bolus insulin adjustments, leading to increased basal insulin doses even after fasting plasma glucose targets have been achieved in an attempt to control postprandial glycemia [2].

Insulin overbasalization not only represents a deviation from recommended practices but may also indicate broader issues in diabetes management, including clinical inertia, the failure to escalate treatment in a timely fashion when therapeutic goals are not met [3]. The phenomenon of clinical inertia, particularly in the context of insulin treatment intensification, has been observed in various settings, including tertiary public diabetes centers with limited pharmacologic options [1]. Given that family medicine residents (FMRs) often serve as primary care providers and play a critical role in managing diabetes care, it is imperative that they are aware of insulin overbasalization and actively combat such inertia to optimize treatment outcomes for their patients [4].

Recent studies have highlighted this issue's complexity. A cross-sectional study by Bieszk et al. [5] demonstrated that overbasalization is a prevalent issue among patients using basal insulin, emphasizing the need for improved education and practice standards. Similarly, a narrative review [6] offered practical guidance on the initiation, titration, and switching of basal insulins, suggesting that there is a recognized need for better-informed strategies among healthcare professionals.

The efficacy and safety of patient-led versus physician-led titration of basal insulin have also been explored in randomized controlled trials [7], which could inform how FMRs empower patients in their care. Furthermore, the role of diabetes self-management education and support, as discussed in the literature [8,9], is essential in equipping both patients and primary care physicians with the skills necessary to manage insulin therapy effectively.

In light of the studies above and the need for improved management practices, this proposal intends to investigate the current state of knowledge, attitudes, and practices (KAP) among FMRs regarding insulin overbasalization. Understanding their level of knowledge, attitudes toward insulin use, and actual practices will help to identify gaps and inform the development of targeted educational interventions to enhance patient care in the context of T2DM management. This initiative will contribute to the global effort in optimizing diabetes care and align with the principles of evidence-based medicine and patient-centered care.

This study aims to assess the level of KAP of FMRs regarding insulin overbasalization in the Family Medicine Academy of King Saud Medical City (KSMC) and to compare the levels of understanding of insulin overbasalization between different levels of residency training program involvement years.

## Materials and methods

Study design

This study utilized a quantitative, cross-sectional approach to comprehensively evaluate the KAP regarding insulin overbasalization among FMRs at KSMC, Riyadh, Saudi Arabia. The research was conducted over 11 months from June 2024 through April 2025, employing a rigorously developed, structured questionnaire administered at a single time point to assess current understanding and behaviors related to this important clinical issue in diabetes management.

Study setting and population characteristics

The investigation was carried out at the Family Medicine Academy of KSMC, a premier tertiary healthcare institution serving as a major referral center in the region. The target population encompassed all actively training FMRs across three postgraduate years (R1-R3), with a total pool of 130 eligible participants as per institutional enrollment records. This specific cohort was selected due to their frontline role in managing diabetic patients in both outpatient and inpatient settings, making their understanding of proper insulin use particularly crucial for patient outcomes.

Inclusion and exclusion criteria

The study employed well-defined participant selection criteria to ensure appropriate representation of the target population. Inclusion required current active enrollment in the family medicine residency program at KSMC during the study period, willingness to voluntarily participate through written informed consent, and physical presence at the institution during data collection phases. Exclusion criteria were systematically applied to remove residents on extended leaves (including maternity, paternity, or medical leave exceeding four weeks), those temporarily assigned to external rotation sites during the study window, and any participants who either declined to participate or submitted substantially incomplete questionnaires (defined as missing >20% of responses).

Sample size determination and statistical justification

The sample size calculation was performed using an established formula for proportion estimation in finite populations, incorporating key parameters from prior research. Based on previous findings indicating an 18% baseline knowledge level about insulin overbasalization among primary care physicians, with a 95% confidence level (Z=1.96) and 7% margin of error, the initial calculation yielded 95 participants [10]. After applying finite population correction for the 130 eligible residents and adding a 15% buffer for potential non-response, the final target sample size was set at 101 participants to ensure adequate statistical power for detecting meaningful differences in knowledge and practice patterns across training levels.

Sampling methodology

A stratified random sampling technique was implemented to guarantee proportional representation across all three residency years (R1, R2, and R3). The sampling frame was constructed using official residency program enrollment lists, with participants randomly selected within each stratum using computer-generated random numbers. This approach ensured balanced representation while maintaining the natural distribution of training levels within the program, enhancing the generalizability of findings to the entire resident population.

Study variables and measurement parameters

The research instrument comprehensively evaluated three primary domains, including knowledge assessment (13 items) covering definitional understanding, recognition of clinical consequences, and identification of at-risk patient populations; attitudinal measurement (18 Likert-scale items) examining perceptions of clinical importance, management confidence, and educational priorities; and practice evaluation (15 items) documenting frequency of patient education, clinical management approaches, and barriers to optimal insulin adjustment.

Ethical considerations

Ethical approval for this study was obtained from the Institutional Review Board of KSMC, under the Ministry of Health in the Kingdom of Saudi Arabia, reference number H1RI-29-May24-01. The study was conducted following the principles outlined in the Declaration of Helsinki, ensuring that the rights and welfare of all participants were protected. Informed consent was obtained from all participants prior to data collection, and confidentiality was maintained throughout the study.

Data collection instrument and validation

The study employed a rigorously developed, self-administered questionnaire collected from literature [4,5,7] that underwent multiple stages of refinement. The instrument was adapted from validated tools used in similar physician knowledge studies. It was further refined through expert panel review (content validity index = 0.89) and pilot testing with 10 residents (Cronbach's alpha = 0.82 for reliability). The final version included demographic items (age, gender, training year), 46 knowledge/attitude/practice items, and five open-ended questions exploring perceived educational needs.

Statistical analysis plan

Data analysis was conducted using SPSS Statistics version 29.0 (IBM Corp. Released 2022. IBM SPSS Statistics for Windows, Version 29.0. Armonk, NY: IBM Corp.), employing both descriptive and inferential statistical methods. Continuous variables were reported as means ± standard deviation, while categorical data were presented as frequencies and percentages. Comparative analyses included independent t-tests and ANOVA for group comparisons, with post-hoc Tukey tests for significant findings. Multivariate logistic regression models were constructed to identify independent predictors of optimal knowledge and practice scores, controlling for demographic and training-level variables. All tests employed a two-tailed significance threshold of p<0.05, with 95% confidence intervals reported for all effect size estimates.

## Results

Our study included 101 FMRs for the assessment of KAP regarding insulin overbasalization (Table 1). Most participants were female (n=55, 54.5%), while males accounted for a slightly lower proportion (n=46, 45.5%). Regarding residency level, the majority were in their first year (R1: n=43, 42.6%), followed by second-year residents (R2: n=37, 36.6%) and third-year residents (R3: n=21, 20.8%). In terms of additional qualifications or experiences, more than half of the residents reported having none (n=59, 58.4%). Pre-career service year experience was noted among 31 residents (30.7%), while a smaller proportion held a postgraduate diploma (n=6, 5.9%) or a master’s degree (n=5, 5.0%).

**Table 1:** Sociodemographic parameters of participants (n=101) N: number, R: residency year

Variable	Category	N (%)
Gender	Female	55 (54.5%)
	Male	46 (45.5%)
Residency year	R1	43 (42.6%)
	R2	37 (36.6%)
	R3	21 (20.8%)
Additional qualifications	None	59 (58.4%)
	Pre-career service year experience	31 (30.7%)
	Master's degree	5 (5.0%)
	Postgraduate diploma	6 (5.9%)

Table 2 shows the assessment of knowledge regarding insulin overbasalization among 101 FMRs. Over half of the participants (n=55, 54.5%) had never heard of insulin overbasalization, while 28 (27.7%) had heard of it but lacked detailed knowledge, and only 18 (17.8%) were familiar with the concept. When asked to identify the correct definition, the majority (n=69, 68.3%) accurately selected "titration of basal insulin beyond the appropriate dose to reach glycemic targets." Regarding potential consequences, most participants identified hypoglycemia (n=78, 77.2%) and weight gain (n=50, 49.5%), followed by blood glucose variability (n=39, 38.6%). Fewer participants recognized other correct consequences, such as increased healthcare utilization (n=22, 21.8%) and cardiovascular disease (n=20, 19.8%). Overall, just over half demonstrated a good knowledge level (n=53, 52.5%), while 39 (38.6%) had average knowledge, and nine (8.9%) had poor knowledge.

**Table 2:** Assessment of knowledge about insulin overbasalization among participants (n=101) N: number

Question	Response option	N (%)
Have you ever heard of the concept of insulin overbasalization?	No (never heard)	55 (54.5%)
	Yes (but don’t know much)	28 (27.7%)
	Yes (familiar with it)	18 (17.8%)
Which of the following best describes insulin overbasalization?	Titration of basal insulin beyond the appropriate dose to reach glycemic targets*	69 (68.3%)
	Use of rapid-acting insulin to cover basal needs	12 (11.9%)
	Insufficient basal insulin leading to high blood sugars	10 (9.9%)
	Skipping mealtime insulin doses and increasing basal insulin	10 (9.9%)
What are the potential consequences of insulin overbasalization? (Multiple responses allowed)	Hypoglycemia	78 (77.2%)
	Weight gain	50 (49.5%)
	Blood glucose variability	39 (38.6%)
	Increased glucose testing requirements	33 (32.7%)
	Quality of life impacts	33 (32.7%)
	Complications of diabetes	28 (27.7%)
	Increased healthcare utilization	22 (21.8%)
	Cardiovascular disease	20 (19.8%)
	Risk of diabetic ketoacidosis	15 (14.9%)
Source of information about insulin overbasalization?	No	52 (51.5%)
	Yes	49 (48.5%)
Overall knowledge level	Poor	9 (8.9%)
	Average	39 (38.6%)
	Good	53 (52.5%)

**Figure 1:**
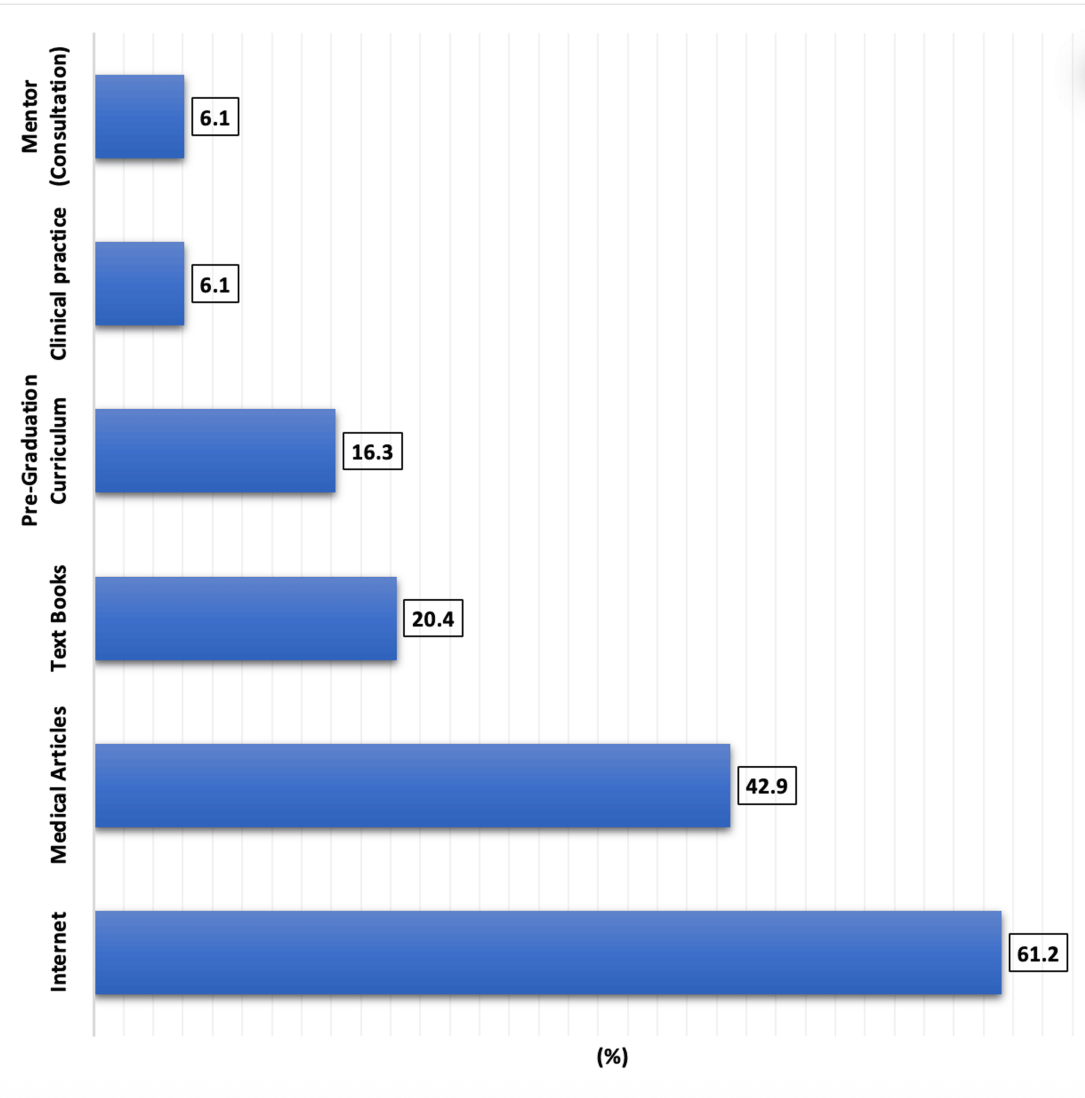
Source of information about insulin overbasalization (n=101)

Figure 1 shows the sources of information about insulin overbasalization among the 101 participants. The most frequently cited source was the Internet (n=62, 61.2%), followed by medical articles (n=43, 42.9%). Fewer participants reported learning from textbooks (n=21, 20.4%) and pre-graduation curriculum (n=17, 16.3%). Notably, only a small fraction acquired knowledge through clinical practice (n=6, 6.1%) or mentor consultation (n=6, 6.1%).

Table 3 shows the participants’ attitudes and practices regarding insulin overbasalization. A vast proportion of the residents (n=46, 45.5%) reported unfamiliarity with recognizing or managing insulin overbasalization in clinical settings. Only a small proportion (n=9, 8.9%) felt fully confident in identifying and managing such cases. Most residents (n=79, 78.2%) had never educated diabetic patients about insulin overbasalization, reflecting limited patient-level engagement. While 46 participants (45.5%) believed this issue falls within the family medicine scope, a notable portion (n=34, 33.7%) were unsure or considered it mainly endocrinology’s domain. Regarding peer and patient communication, 41 (40.6%) acknowledged their unfamiliarity and learning needs. Encouragingly, 33 (32.7%) expressed strong motivation to learn more within the next month, and 30 (29.7%) planned to read on the topic within six months. Overall, 53 (52.5%) displayed a good attitude toward practice, while 48 (47.5%) showed poor attitude levels.

**Table 3:** Assessment of attitude and practice on insulin overbasalization among participants (n=101)

Question	Response option	N (%)
Do you know how to identify and recognize patients with signs of insulin overbasalization?	Not familiar	46 (45.5%)
	Not very confident – need more training	24 (23.8%)
	Somewhat confident – may consult specialists	22 (21.8%)
	Fully confident	9 (8.9%)
Have you ever educated diabetic patients about insulin overbasalization and its consequences?	No	79 (78.2%)
	Yes	22 (21.8%)
In your opinion, does the identification and management of insulin overbasalization fall within the scope of practice for family medicine?	Not sure/mainly endocrinology	34 (33.7%)
	Definitely within family medicine scope	46 (45.5%)
	Somewhat – need collaboration	21 (20.8%)
Which statement best describes your attitude toward sharing the concept of insulin overbasalization with your colleagues and patients?	I am not familiar with the concept of insulin overbasalization and need to learn more about it	41 (40.6%)
	I have heard of insulin overbasalization, but do not fully understand it or feel adequately equipped to educate patients about it	13 (12.9%)
	I am somewhat familiar with the concept of insulin overbasalization, but would benefit from further education to feel confident in managing it	28 (27.7%)
	I have a good understanding of insulin overbasalization, and I am comfortable discussing it and its potential consequences with patients.	10 (9.9%)
	I am highly knowledgeable about insulin overbasalization and actively educate others on its implications and management.	9 (8.9%)
Please select the statement that most accurately reflects your attitude to enhancing your learning needs about insulin overbasalization	I currently have no plans to educate myself on insulin overbasalization further, as I do not see it as relevant to my practice at this time	11 (10.9%)
	While I understand that knowledge of insulin overbasalization is beneficial, I have not yet planned a specific timeframe to learn more about it	6 (5.9%)
	I am interested in learning more about insulin overbasalization and aim to read about it in the next six months	30 (29.7%)
	I recognize the importance of understanding insulin overbasalization and plan to educate myself on the subject within the next three months	21 (20.8%)
	I am highly motivated to expand my knowledge on insulin overbasalization and intend to prioritize learning about it within the next month	33 (32.7%)
Overall attitude toward insulin overbasalization practice	Poor	48 (47.5%)
	Good	53 (52.5%)

**Figure 2:**
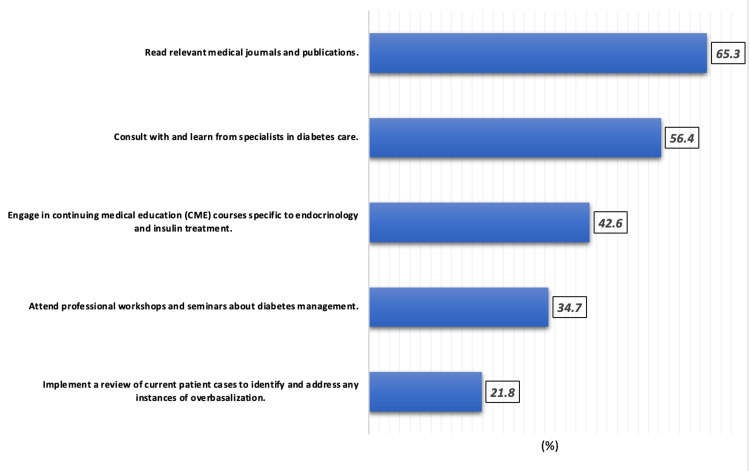
Plans to enhance awareness of insulin overbasalization (n=101)

Figure 2 shows the participants’ plans to enhance their awareness and understanding of insulin overbasalization. The most commonly selected approach was reading relevant medical journals and publications (n=66, 65.3%), followed by consulting with specialists in diabetes care (n=57, 56.4%). A considerable proportion also expressed interest in continuing medical education (CME) courses focused on endocrinology and insulin treatment (n=43, 42.6%). Fewer participants planned to attend professional workshops or seminars (n=35, 34.7%), and the least preferred method was to implement a review of current patient cases to identify and manage overbasalization (n=22, 21.8%).

Table 4 shows the association between participant characteristics and their knowledge and attitude/practice scores related to insulin overbasalization. While knowledge scores increased progressively with residency level, R1 (mean = 4.58 ± 2.25), R2 (5.24 ± 2.03), and R3 (5.71 ± 2.49), the difference was not statistically significant (p = 0.137). However, a significant association was observed for attitude and practice scores across residency years (p = 0.023), with R3 residents scoring the highest (mean = 7.67 ± 3.10), indicating more confident and informed practices. Gender differences in both knowledge (p = 0.240) and attitude/practice (p = 0.408) were not statistically significant, though males had slightly higher mean scores in both domains. Participants without additional qualifications scored higher in knowledge (5.31 ± 2.43) compared to those with diplomas or master’s degrees, but this trend lacked statistical significance.

**Table 4:** Association of different features with knowledge and attitude practice score (n=101) (±) independent t-test, (±±) ANOVA, p-value is considered significant when <0.05 SD: standard deviation, ANOVA: analysis of variance

Variable	Category	Knowledge score mean ± SD	p-value	Attitude and practice score mean ± SD	p-value
Residency year	R1	4.58 ± 2.25	0.137±	5.19 ± 3.57	0.023±
	R2	5.24 ± 2.03		5.86 ± 3.16	
	R3	5.71 ± 2.49		7.67 ± 3.10	
Gender	Female	4.82 ± 2.33	0.240±±	5.69 ± 3.25	0.408±±
	Male	5.35 ± 2.13		6.26 ± 3.65	
Additional qualifications	None	5.31 ± 2.43	0.118±	6.08 ± 3.63	0.423±
	Pre-career service year exp.	5.13 ± 2.06		6.13 ± 2.96	
	Master's degree	3.80 ± 0.45		6.00 ± 4.53	
	Postgraduate diploma	3.33 ± 1.03		3.67 ± 2.58	

Table 5 shows the adjusted logistic regression analysis identifying predictors of good knowledge and positive attitude/practice toward insulin overbasalization among FMRs. Regarding the adjusted predictors of high knowledge, although the male residents had higher odds of demonstrating high knowledge (OR = 2.05, 95% CI: 0.89-4.74), the result was not statistically significant (p = 0.093). Similarly, a higher residency year was associated with increased odds of better knowledge (OR = 1.48, 95% CI: 0.86-2.54), but again, without statistical significance (p = 0.153). Additional qualifications showed a non-significant inverse association (OR = 0.63, p = 0.273), suggesting no added knowledge benefit. For good attitude and practice, a statistically significant association was found for residency year (p = 0.043), with higher levels of training predicting better attitudes and practices (OR = 1.74, 95% CI: 1.02-2.99). Gender and qualifications did not significantly predict good practice, though males had higher odds (OR = 1.52, p = 0.328).

**Table 5:** Adjusted predictors of high knowledge and good attitude practice toward insulin overbasalization p-value is considered significant when <0.05 B: regression coefficient, OR: odds ratio, CI: confidence interval

Model and predictors	B	p-value	OR	95% CI
Good knowledge				
Gender (male)	0.719	0.093	2.052	0.888-4.743
Higher residency year	0.393	0.153	1.481	0.864-2.540
Additional qualification	-0.470	0.273	0.625	0.270-1.449
Constant	-0.725	0.245	0.485	
Positive attitude/practice				
Gender (male)	0.417	0.328	1.518	0.658-3.503
Higher residency year	0.555*	0.043*	1.742*	1.017-2.985*
Additional qualification	0.334	0.440	1.396	0.599-3.254
Constant	-1.211	0.054	0.298	

**Figure 3:**
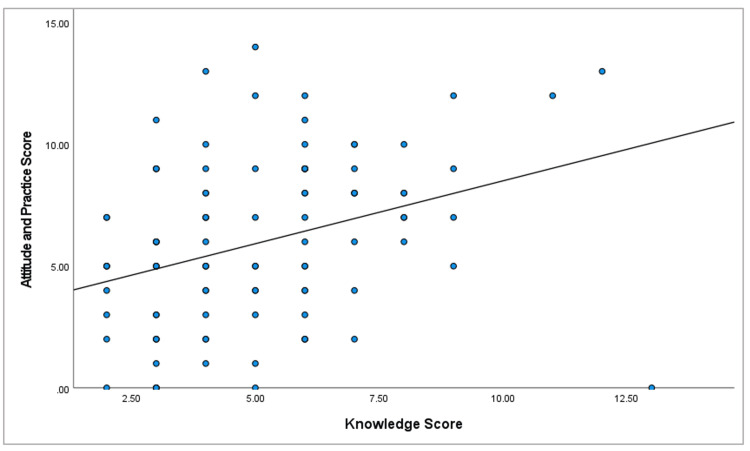
Scatter plot showing the correlation between knowledge and attitude practice toward insulin overbasalization

Table 6 and Figure 3 show the correlation between knowledge and attitude/practice scores toward overbasalization of insulin among the 101 FMRs. A statistically significant moderately strong positive correlation was observed (r = 0.339, p = 0.001), indicating that residents with higher knowledge scores tended to exhibit better attitudes and practices regarding insulin overbasalization. This relationship underscores the impact of knowledge on shaping clinical behavior and highlights the need for targeted educational strategies to enhance both understanding and practice among family medicine trainees.

**Table 6:** Correlation between knowledge and attitude practice toward insulin overbasalization

		Attitude and practice score
Knowledge score	Pearson correlation	0.339
	Sig. (2-tailed)	0.001
	N	101

## Discussion

T2DM is a condition that often requires insulin therapy for its management, with the basal insulin commonly used for initial control [9]. However, the improper titration of insulin can lead to insulin overbasalization with excessive reliance on basal insulin despite achieving fasting plasma glucose targets, neglecting postprandial control [10]. The residents of the family medicine play a crucial role in the management of diabetic patients; thus, this study aims to assess their KAP regarding the insulin overbasalization.

This study shows that more than half of the respondents (54.5%) had never heard of the term insulin overbasalization, and only 17.8% reported familiarity with the concept. These results are consistent with findings from prior literature indicating that overbasalization is often underrecognized in primary care settings and is a frequently overlooked barrier to achieving optimal glycemic targets [11]. A U.S.-based study shows that approximately 40% of patients on basal insulin were at risk of overbasalization, yet few primary care physicians could define or detect it systematically [12]. Moreover, Davies et al. show that many providers over-titrate basal insulin despite postprandial hyperglycemia being the issue, reflecting poor titration practices. They suggested using basal insulin plus DPP-4 inhibitors as an effective and safe alternative in older adults [13].

Notably, in our cohort, although 68.3% correctly identified the definition of insulin overbasalization, their source of information was predominantly informal, with the Internet (61.2%) and medical articles (42.9%) being the top sources. Alarmingly, structured educational routes such as pre-graduation curricula (16.3%), clinical practice (6.1%), and mentorship (6.1%) were minimally reported. These findings suggest a curriculum deficit, aligning with the previous literature emphasizing the underrepresentation of advanced insulin management topics in undergraduate and early residency training. For instance, a study by Alhagawy et al. reported that only 50% of medical professionals felt inadequately trained to manage complex insulin regimens, often defaulting to endocrinology referrals instead [14].

Moreover, the attitude and practice findings further underline this knowledge gap. Nearly half of the participants (45.5%) did not know how to identify or manage overbasalization, and only 8.9% felt fully confident. Patient education was notably lacking, with 78.2% reporting they had never informed diabetic patients about this risk, which is a concerning trend given the potential consequences of overbasalization, including hypoglycemia, weight gain, glucose variability, and increased healthcare utilization. These findings align with international data suggesting that therapeutic or clinical inertia and lack of confidence among primary care providers contribute to the under-recognition and under-treatment of insulin-related complications [15].

Interestingly, this study also shows that the attitude and practice scores were significantly associated with residency year (p = 0.023), with third-year residents showing higher confidence and engagement. This progression reflects the expected clinical maturity gained through experience, yet also signals the missed opportunity for earlier intervention. In contrast, knowledge scores did not significantly differ by year (p = 0.137), suggesting that practical exposure rather than formal instruction plays a more critical role in shaping insulin management behavior. This trend is consistent with previous literature emphasizing the importance of experiential learning and case-based mentoring in bridging theoretical gaps [16].

While gender and additional qualifications did not significantly impact KAP scores, logistic regression analysis revealed that higher residency year was a significant independent predictor of good practice and positive attitude (OR = 1.74, p = 0.043). This finding reinforces the need to integrate overbasalization awareness into earlier stages of training to avoid over-reliance on trial-and-error learning. Notably, no benefit was observed from holding a master's degree or postgraduate diploma, indicating a disconnect between advanced academic credentials and practical competency in this area.

Notably, this study also shows that a moderately strong positive correlation was observed between knowledge and attitude/practice scores (r = 0.339, p = 0.001), suggesting that enhancing knowledge can positively influence clinical behavior. This is in line with behavior change models in medical education, where knowledge often catalyzes attitude shift and practice adaptation [17]. It also reinforces the importance of targeted CME interventions. In our sample, residents reported plans to improve their knowledge primarily through reading journals (65.3%) and consulting specialists (56.4%), while fewer opted for active interventions such as patient case reviews (21.8%) or workshops (34.7%). These findings indicate a preference for passive learning, which has been shown in past studies to be less effective than interactive, scenario-based learning formats [18].

Limitations of the study

There are several limitations of this study. This study was conducted at a single center, which limits the generalizability of its findings to other institutions or regions. The sample size is inadequate and may not capture all variability in knowledge and practices among FMRs nationwide. Additionally, the use of the self-administered questionnaire may introduce response bias, with participants potentially overestimating their knowledge or attitudes. The cross-sectional design restricts causal interpretations between knowledge and practice. Lastly, the study did not assess actual clinical behavior, relying instead on self-reported practices, which may differ from real-world application.

Implications and future direction

There are critical gaps in the knowledge and the clinical confidence of FMRs regarding insulin overbasalization, which emphasizes the need for structured educational interventions. Thus, incorporating insulin titration principles into residency training and CME programs may improve patient safety and glycemic control. The future efforts should focus on longitudinal studies assessing changes in clinical behavior post-intervention and expanding the research to multiple centers for broader applicability. Additionally, integrating case-based simulations and mentorship from endocrinologists could enhance practical skills, reduce clinical insufficiency, and foster a more proactive approach to insulin management in primary care.

## Conclusions

This study shows the significant weaknesses in KAP regarding insulin overbasalization among FMRs at KSMC. Over half were unfamiliar with the concept, and few residents felt confident identifying or managing it clinically. Knowledge levels were moderately associated with improved attitudes and practices, and higher residency year was a significant predictor of better performance. However, additional qualifications did not enhance outcomes. These findings underscore the need for targeted educational interventions early in training to improve insulin management competencies and reduce clinical inertia in diabetes care.

## Appendices

**Table 7:** Questionnaire on KAP regarding insulin overbasalization KAP: knowledge, attitude, and practice

Section	Question Number	Question	Response Options
A. Sociodemographic Data	1	Current residency year:	☐ R1 ☐ R2 ☐ R3
	2	Gender:	☐ Male ☐ Female
	3	Additional qualifications/experiences:	☐ Master's degrees ☐ Postgraduate diplomas ☐ Pre-career service year experience ☐ Other: ___________
B. Knowledge	1	Have you heard of insulin overbasalization?	☐ Yes, familiar ☐ Heard but don't know much ☐ Never heard
	2	Source of information (if yes):	☐ Medical Articles ☐ Textbooks ☐ Pre-graduate curriculum ☐ Internet ☐ Other: ___________ ☐ Never heard
	3	Best description of overbasalization:	☐ A. Insufficient basal insulin ☐ B. Rapid-acting for basal needs ☐ C. Titration beyond appropriate dose ☐ D. Skipping mealtime for more basal
	4	Potential consequences (select all):	☐ Hypoglycemia ☐ Weight Gain ☐ Cardiovascular disease ☐ Blood Glucose Variability ☐ Risk of DKA ☐ Quality of Life Impacts ☐ Increased Healthcare Utilization ☐ Diabetes Complications ☐ More glucose testing
C. Attitude and Practice	1	Confidence in identifying cases:	☐ A. Fully confident ☐ B. Somewhat confident ☐ C. Need more training ☐ D. Would refer ☐ E. Unfamiliar
	2	Have you educated patients?	☐ Yes ☐ No
	3	Scope of practice opinion:	☐ A. Definitely FM scope ☐ B. Needs endo collaboration ☐ C. Endo should manage ☐ D. Unsure
	4	Plans to enhance knowledge:	☐ A. Workshops/seminars ☐ B. CME courses ☐ C. Medical journals ☐ D. Consult specialists ☐ E. Case reviews
	5	Attitude toward sharing knowledge:	☐ A. Highly knowledgeable educator ☐ B. Comfortable discussing ☐ C. Need more education ☐ D. Don't understand well ☐ E. Unfamiliar
	6	Learning motivation:	☐ A. Prioritize within month ☐ B. Plan within 3 months ☐ C. Aim within 6 months ☐ D. No timeframe ☐ E. No plans

## References

[1] Alvarenga MA, Komatsu WR, de Sa JR, Chacra AR, Dib SA (2018). Clinical inertia on insulin treatment intensification in type 2 diabetes mellitus patients of a tertiary public diabetes center with limited pharmacologic armamentarium from an upper-middle income country. Diabetol Metab Syndr.

[2] Baskaran C, Volkening LK, Diaz M, Laffel LM (2015). A decade of temporal trends in overweight/obesity in youth with type 1 diabetes after the diabetes control and complications trial. Pediatr Diabetes.

[3] Baxter M, Morimoto Y, Tamiwa M, Hattori M, Peng XV, Lubwama R, Maegawa H (2020). A real-world observational study evaluating the probability of glycemic control with basal insulin or glucagon-like peptide-1 receptor agonist in Japanese patients with type 2 diabetes. Diabetes Ther.

[4] Bieszk N, Grabner M, Wei W (2017). Personalized care and the role of insulin as a vehicle to optimizing treatments in diabetes care. J Manag Care Spec Pharm.

[5] Bieszk N, Reynolds SL, Wei W, Davis C, Kamble P, Uribe C (2016). “Act on threes” paradigm for treatment intensification of type 2 diabetes in managed care: results of a randomized controlled study with an educational intervention targeting improved glycemic control. J Manag Care Spec Pharm.

[6] Castellana M, Procino F, Sardone R, Trimboli P, Giannelli G (2020). Efficacy and safety of patient-led versus physician-led titration of basal insulin in patients with uncontrolled type 2 diabetes: a meta-analysis of randomized controlled trials. BMJ Open Diabetes Res Care.

[7] Chertok Shacham E, Kfir H, Schwartz N, Ishay A (2018). Glycemic control with a basal-bolus insulin protocol in hospitalized diabetic patients treated with glucocorticoids: a retrospective cohort study. BMC Endocr Disord.

[8] Choe EY, Lee YH, Lee BW, Kang ES, Cha BS, Lee HC (2012). Glycemic effects of once-a-day rapid-acting insulin analogue addition on a basal insulin analogue in Korean subjects with poorly controlled type 2 diabetes mellitus. Diabetes Metab J.

[9] Coetzee A (2023). An introduction to insulin use in type 2 diabetes mellitus. S Afr Fam Pract (2004).

[10] Chan SP, Aamir AH, Bee YM (2022). Practical guidance on basal insulin initiation and titration in Asia: a Delphi-based consensus. Diabetes Ther.

[11] Cowart K, Franks R, Pane O, Murphy E, Oldziej K (2022). Addressing overbasalization to achieve glycemic targets. ADCES Pract.

[12] Stewart-Lynch A, Meyers R, Sidig D, McConville SO, Heiple L (2024). Quantifying and characterizing the presence of insulin overbasalization in a family medicine practice. Clin Diabetes.

[13] Davies MJ, Aroda VR, Collins BS (2022). Management of hyperglycaemia in type 2 diabetes, 2022. A consensus report by the American Diabetes Association (ADA) and the European Association for the Study of Diabetes (EASD). Diabetologia.

[14] Alhagawy AJ, Yafei S, Hummadi A (2022). Barriers and attitudes of primary healthcare physicians to insulin initiation and intensification in Saudi Arabia. Int J Environ Res Public Health.

[15] Zhu NA, Harris SB (2020). Therapeutic inertia in people with type 2 diabetes in primary care: a challenge that just won’t go away. Diabetes Spectr.

[16] Mai Phuc T (2025). Bridging theory and practice: the role of experiential learning and mentorship in enhancing the transition from university to high-school teaching for pre-service English teachers. J Teach Learn.

[17] Singh D, Biju B, Kumar L, Arya S, Singh AK (2023). Assessing the impact of health education on health behavior change. J Chem Health Risks.

[18] Ang KCS, Afzal F, Crawford LH (2021). Transitioning from passive to active learning: preparing future project leaders. Proj Leadersh Soc.

